# Synthesis and Investigation of CuGeO_3_ Nanowires as Anode Materials for Advanced Sodium-Ion Batteries

**DOI:** 10.1186/s11671-018-2609-z

**Published:** 2018-07-04

**Authors:** Lin Fu, Xueying Zheng, Lanyan Huang, Chaoqun Shang, Ke Lu, Xuzi Zhang, Benben Wei, Xin Wang

**Affiliations:** 1 National Center for International Research on Green Optoelectronics, South China Normal University, Guangzhou, 510006 China; 2 School of Materials Science and Engineering, Tongji University, Shanghai, 201804 China; 3 International Academy of Optoelectronics at Zhaoqing, South China Normal University, Zhaoqing, 526238 China

**Keywords:** Sodium-ion batteries, Ternary compound, CuGeO_3_ nanowires, Anode material

## Abstract

Germanium is considered as a potential anode material for sodium-ion batteries due to its fascinating theoretical specific capacity. However, its poor cyclability resulted from the sluggish kinetics and large volume change during repeated charge/discharge poses major threats for its further development. One solution is using its ternary compound as an alternative to improve the cycling stability. Here, high-purity CuGeO_3_ nanowires were prepared via a facile hydrothermal method, and their sodium storage performances were firstly explored. The as-obtained CuGeO_3_ delivered an initial charge capacity of 306.7 mAh g^−1^ along with favorable cycling performance, displaying great promise as a potential anode material for sodium ion batteries.

## Background

In the past two decades, lithium-ion batteries (LIBs) have successfully dominated the market in the field of energy storage and conversion [1, 2]. LIBs are now serving as the power source for a variety of devices, ranging from smartphones to electric vehicles (EVs) [3–7]. However, future development of LIBs is greatly hindered by the shortage of lithium resources which inevitably limits their large-scale application [8]. Hence, seeking other alternatives to replace lithium is of vital significance. Based on the earth-abundant and similar physical and chemical characteristics with lithium, sodium proves one of the most promising candidates in rechargeable batteries [9]. Over the past years, a significant progress of sodium-ion batteries (SIBs) for cathode materials has been obtained by drawing the experience from LIB systems [9–11]. While potential materials for the anode side still remain underdeveloped. It is generally known that the size of sodium ion is significantly larger than that of lithium ion, which leads to sluggish electrochemical reaction kinetics and large volume change accompanied by unstable solid electrolyte interphase (SEI) layer, resulting in inferior cycling stability and rate capability of SIBs [12]. Thus, seeking potential candidates for the anode is particularly important but challenging.

Germanium (Ge) as an anode material has been extensively investigated for SIBs owing to their high theoretical specific capacities (369 mAh g^−1^ based on NaGe) [13]. Nevertheless, it is interesting that elemental Ge displays fascinating capacities only in the thin film and amorphous structure electrodes [14]. In order to improve electrochemical properties for coarser structures, one feasible strategy is to introduce carbonaceous materials. For example, Yin and co-workers designed and synthesized hollow carbon boxes/Ge hybrid material as the anode in SIBs and obtained high reversible capacity even after 500 cycles, which approximated its theoretical value [15]. Another successful method is to use binary or ternary Ge-based compounds with nanostructure. Binary or ternary compounds incorporated with carbonaceous materials have been reported to deliver a greatly improved cycling and rate performances as compared to single Ge [16–18]. Based on the experimental results in LIBs, it is worth noting that ternary compounds exhibit excellent electrochemical properties due to the formation of the intermediate products during the discharge process, which serve as an inert matrix to mitigate the volume changes and prevent the agglomeration of active material particles [19]. Importantly, the intermediate products of ternary Ge-based compounds include amorphous Ge, which is reported to improve sodiation kinetics [14, 20]. CuGeO_3_ (CGO) is a typical I-V-VI ternary Ge-based oxide and exhibits superior lithium storage performance [21]. Based on the assumption of seven Na^+^ reaction calculated, the theoretical specific capacity of CGO is 1018 mAh g^−1^. However, the sodium storage property of CGO is rarely explored to date.

In this work, CGO nanowire was successfully synthesized by a facile and reliable hydrothermal reaction and was firstly explored as an anode material for its sodium storage performance. It exhibits excellent electrochemical performances in terms of reversible capacity, coulombic efficiency (CE), cycling stability, and rate property, which are greatly improved in comparison to that of elemental Ge. The results indicate that using ternary compounds is one of the most effective approaches to promote the study of Ge-based anode material for SIBs.

## Methods

### Material Preparation

CGO nanowires were prepared via a facile hydrothermal method. First of all, 0.1 g cetyltrimethylammonium bromide (CTAB) was added into a 15-mL distilled water to form homogeneous solution under magnetic stirring for 1 h at room temperature. Next, 5 mM Cu(CH_3_COO)_2_·H_2_O and 5 mM GeO_2_ were added to the above solution, respectively, and the mixed solution was stirred continuously for 1 h. After that, the reaction mixture was loaded and sealed into a Teflon-lined stainless-steel autoclave with 20 mL inner volume and heated at 180 °C for 24 h before cooling down to room temperature. Last, the CGO nanowires were collected by washing with distilled water and ethanol for three times and dried at 60 °C for 24 h in an oven. The Ge materials were prepared by high-energy ball milling of crystalline Ge powders (Alfa Aesar).

### Material Characterization

X-ray diffraction (XRD) details of the samples were collected on a Bruker-AXS Micro-diffractometer (D8 ADVANCE) under CuKα radiation (*λ* = 1.5406 Å) at a voltage of 30 kV. The microstructure images of the samples were acquired on a HITACHI S-4800 field emission scanning electron microscopy (SEM) and a HITACHI H-7650 transmission electron microscopy (TEM). The selected area electronic diffraction (SAED) patterns were obtained using a JEM 2100HR TEM.

### Electrochemical Measurements

For the working electrode preparation, 80 wt% of CGO nanowires, 10 wt% of Super P carbon, and 10 wt% poly(acrylic) acid binder were mixed with appropriate amount of distilled water to form a slurry and then uniformly casted onto a copper foil. Afterwards, the electrodes were dried in vacuum oven at 60 °C for 24 h for moisture removing. The Ge electrode was prepared via similar processes. The electrolyte consisted of 1 M NaClO_4_ salt dissolved in ethylene carbonate/dimethyl carbonate (EC/DMC, 1:1 *v*/*v*) with 5 vol% fluoroethylene carbonate (FEC) as additive. The working electrodes were assembled into coin-type (CR2032) cells in an argon-filled glove box with glass microfiber filter and Na metal as separator and counter electrode, respectively, and appropriate amount of above electrolyte. The electrochemical measurements were evaluated by cyclic voltammetry (CV, CHI 660B electrochemical workstation) and galvanostatic charge/discharge tests (LAND 2001A Battery Tester) in the voltage range of 0.05–2.0 V vs. Na/Na^+^. The weight loading of CGO active material in working electrode was ca. 1.0 mg cm^−2^, and the specific capacity was calculated based on the active material.

## Results and Discussion

**Fig. 1:**
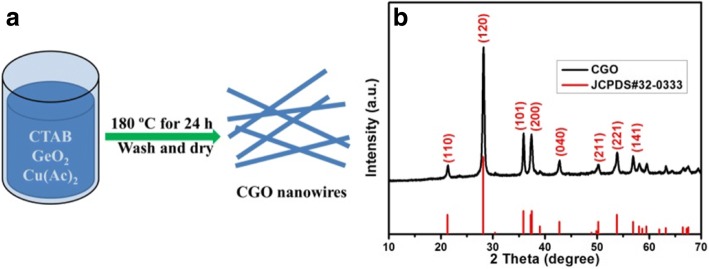
**a** Schematic illustration of the preparation and **b** XRD pattern of the CGO nanowires

A schematic illustration of the preparation process of the CGO nanowires is displayed in Fig. 1a. The homogeneous solution was formed by mixing the CTAB, GeO_2_, and Cu(CH_3_COO)_2_·H_2_O with appropriate amount of distilled water. Among them, CTAB was used as a surfactant. After 24 h, the CGO nanowires were produced under the hydrothermal environment. At the hydrothermal process, the starting material GeO_2_ can be dissolved in water to give H_2_GeO_3_ [22]. Subsequently, H_2_GeO_3_ reacted with Cu(CH_3_COO)_2_·H_2_O to form orthorhombic CGO [23]. Based on the above discussion with nucleation mechanism [24], a possible synthesis mechanism for the CGO nanowires is proposed to be expressed such as:

$$ {\displaystyle \begin{array}{l}{\mathrm{GeO}}_2+{\mathrm{H}}_2\mathrm{O}\to {\mathrm{H}}_2{\mathrm{GeO}}_3\\ {}\mathrm{Cu}{\left({\mathrm{CH}}_3\mathrm{COO}\right)}_2+{\mathrm{H}}_2{\mathrm{GeO}}_3\to {\mathrm{CuGeO}}_3+2{\mathrm{CH}}_3\mathrm{COO}\mathrm{H}\end{array}} $$

XRD pattern was used to confirm the crystal structures and chemical composition of the as-prepared samples. As depicted in Fig. 1b, all the peaks of XRD spectrum are well-matched with the standard JCPDS card (No. 32-0333) without peaks of impurities, which can be concluded that the as-synthesized CGO nanowire is pure phase. The 2*θ* peaks at 21.238°, 28.09°, 35.787°, 37.408°, etc. are attributed to (110), (120), (101), (200), etc. lattice planes of orthorhombic phase, respectively. In addition, the strong diffraction peaks indicate good crystallinity of the products.

**Fig. 2:**
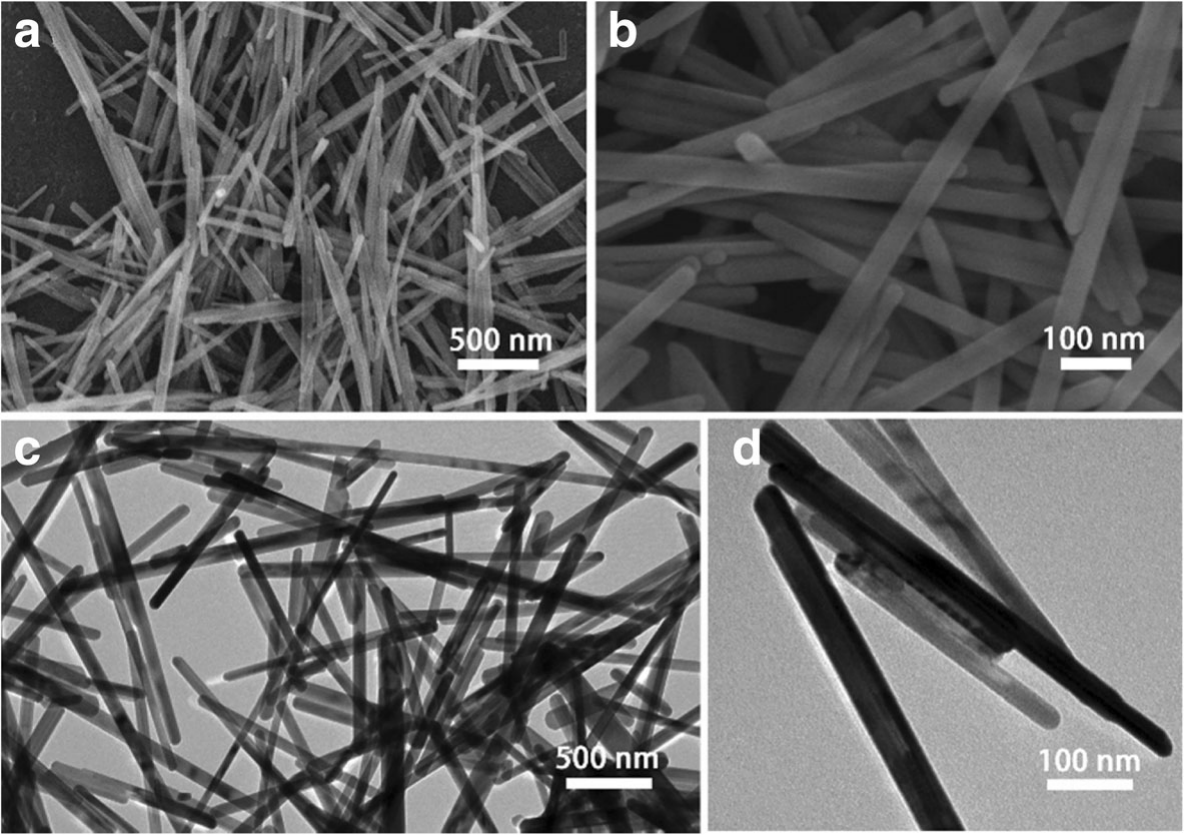
**a**, **b** SEM and **c**, **d** TEM images of the CGO nanowires

The SEM and TEM images were employed to observe the morphology of these hydrothermal products. As displayed in SEM image (Fig. 2a), the as-obtained CGO are uniform nanowires with a length more than 1 μm, which agrees well with the reported result [25]. The high-magnification SEM image (Fig. 2b) reveals that the average diameter of CGO nanowires is about 20 nm. The TEM images are displayed in Fig. 2c, d; it can be clearly seen that the microstructure of CGO nanowires is consistent with the above SEM results. The nanostructured anode materials have been demonstrated to improve electrochemical performances owing to their large surface area and reduced diffusion pathway [26]. The high uniformity nanowire is beneficial to accommodate volume changes and enhance sodium-ion diffusion in active materials during charge/discharge processes [27].

**Fig. 3:**
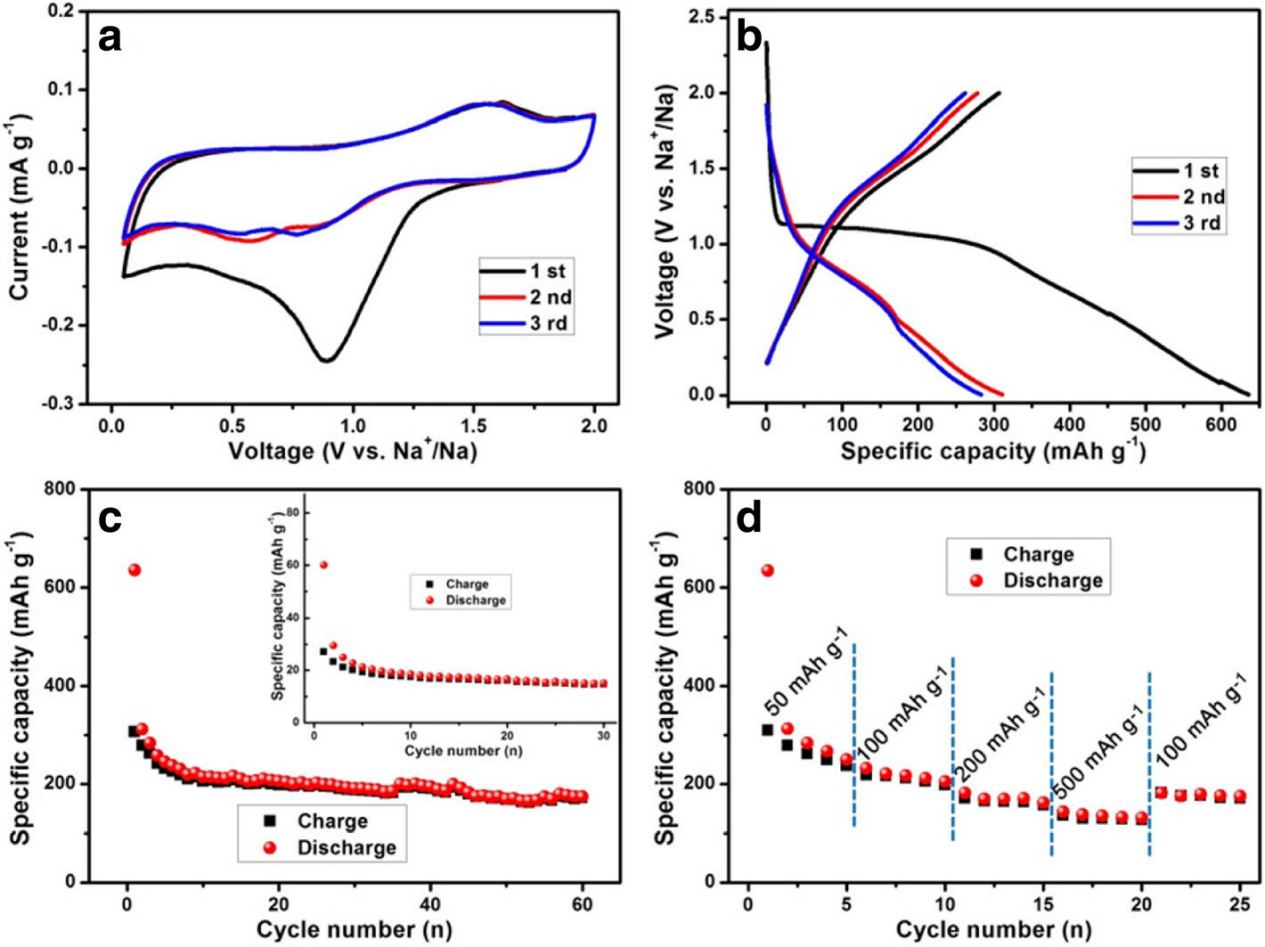
**a** The initial three CV curves of CGO nanowires at a scanning rate of 0.2 mV s^−1^. **b** The initial three charge/discharge curves and **c** cycling performance of the CGO nanowires at a current density of 50 mA g^−1^. Inset in **c** is the cycling performance of the elemental Ge at a current density of 50 mA g^−1^. **d** Rate capability of CGO nanowires at different current densities (from 50 to 500 mA g^−1^)

**Fig. 4:**
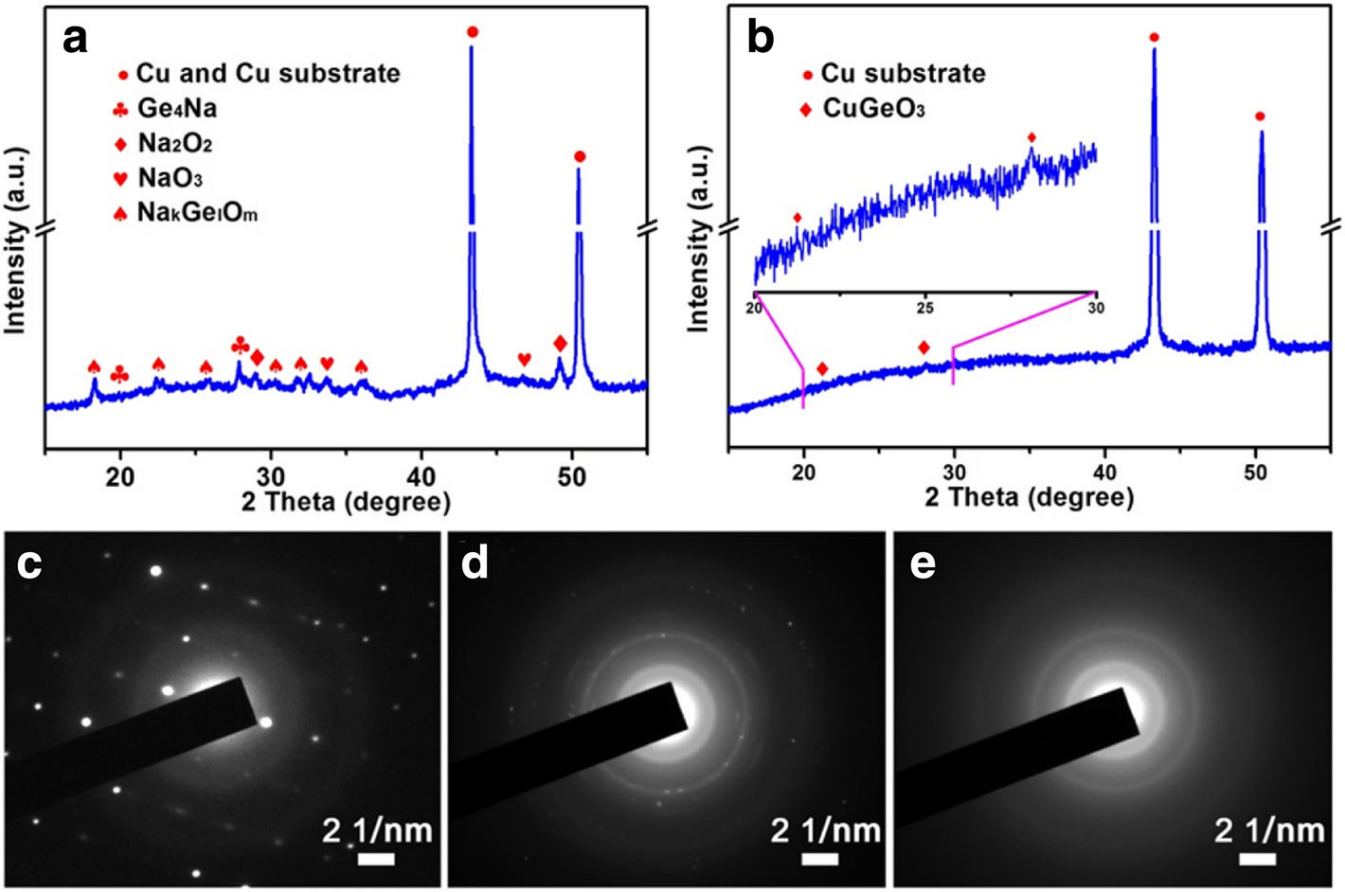
Ex situ XRD patterns of the CGO electrode when **a** discharged to 0.05 V and **b** charged to 2.0 V. **c** SAED pattern of the CGO samples. SAED patterns of the CGO electrode when **d** discharged to 0.05 V and **e** charged to 2.0 V

To explore the sodium storage characteristics of the CGO nanowires, a series of electrochemical measurements were performed. CV is an effective route to evaluate the reaction mechanism during the sodiation/desodiation process. Figure 3a illustrates typical CV curves of the CGO anode material with a scan rate of 0.2 mV s^−1^ in the voltage window of 0.05–2.0 V (vs. Na/Na^+^). The first cathodic scan shows a broad and strong peak located at 0.8 V, obviously different from the later cycles, which can be attributed to the multi-step conversion of CGO to produce Cu, Ge, Na_*x*_O_*y*_, Na_*k*_Ge_*l*_O_*m*_, and irreversible decomposition of electrolyte to form SEI layer [17, 28]. This peak separated into two peaks and transferred to at about 0.6 and 0.75 V in the subsequent cycles, which could be assigned to the decrease of irreversible reaction and the stabilization of as-formed SEI layer. Similar phenomena were reported for the ternary anode materials [29]. The reduction peak at the voltage of around 0.01 V is ascribed to the alloying of Na_*z*_Ge, and the oxidation peak at about 0.2 V corresponds to the reversible de-alloying of Na_*z*_Ge [30]. The anodic peak loaded at 1.5 V represented the further oxidation of discharge products. Phase changes of CGO electrode were investigated to further explore sodium storage mechanism, and the ex situ XRD measurement was performed on the first discharged and charged products. Figure 4a shows the XRD patterns of CGO electrode discharged at 0.05 V, all the peaks of CGO disappeared completely, and some new peaks of Cu, Ge_4_Na, Na_2_O_2_, NaO_3_, and Na_*k*_Ge_*l*_O_*m*_ (such as Na_4_GeO_4_, Na_2_Ge_2_O_5_, Na_6_Ge_2_O_7_) appeared, indicating that CGO reacted with Na during the discharge process. Note that the reflection peaks of Na_*k*_Ge_*l*_O_*m*_ were clearly found, which could be assigned to the unique crystal structure orthorhombic CGO. The orthorhombic CGO was structured by the corner-sharing GeO_4_ tetrahedra as basic building blocks and Cu^2+^ as a junction to form chains along the *c*-axis [25]. Each Cu atom was assigned to form strongly deformed CuO_6_ octahedron with surrounding six O atoms. When charged to 2.0 V (Fig. 4b), the whole diffraction peaks became indistinct, except Cu substrate, and two weak peaks can be well indexed to CGO, indicating that the recovered CGO is of poor crystallinity or amorphous. This result was confirmed by the SAED patterns of pristine CGO and discharged and charged products (Fig. 4c, d). Interestingly, these poor crystallinity or amorphous products are beneficial for subsequent solid state diffusion of Na^+^ [12]. Based on the above results and discussion, we propose that the sodium storage process of CGO is attributed to the combination of conversion and alloy reaction such as:

$$ {\displaystyle \begin{array}{l}{\mathrm{CuGeO}}_3+{\mathrm{Na}}^{+}\to \mathrm{Cu}+\mathrm{Ge}+{\mathrm{Na}}_x{\mathrm{O}}_y+{\mathrm{Na}}_k{\mathrm{Ge}}_l{\mathrm{O}}_m\\ {}\mathrm{Ge}+{\mathrm{Na}}^{+}\to {\mathrm{Na}}_z\mathrm{Ge}\end{array}} $$

The integrated areas of the second and third CV curves are almost the same, indicative of good reversibility after initial cycle.

The electrochemical performances were further investigated via galvanostatic charge/discharge cycling measurements under the same voltage range. The cycling performance of elemental Ge anode materials at a current density of 50 mA g^−1^ is inset in Fig. 3c, the initial charge/discharge capacity was 27.1/60.1 mAh g^−1^ (CE of 45.09%), which is significantly lower than that of the theoretical value. Moreover, the retained capacity was only 15 mAh g^−1^ after 30 cycles. It is reported that the sluggish sodiation kinetics of Ge is the direct reason why using amorphous structure materials is successful in obtaining high specific capacity [14]. Importantly, CGO were found to form amorphous Ge, which can be homogeneously distributed in the Cu and Li_2_O matrix before the alloy reaction during each discharge process [20, 31, 32]. Figure 3b shows the initial three charge/discharge curves of CGO nanowires at a current density of 50 mA g^−1^. All the voltage plateaus corresponded well to the above CV results.

Cycling performance and rate capability are the two main issues to evaluate the sodium storage characteristics of CGO as an anode material. As depicted in Fig. 3c, the CGO nanowires delivered an initial charge capacity of up to 306.7 mAh g^−1^ and an initial CE of 61.74% at a constant current density of 50 mA g^−1^. The high capacity loss in the initial cycle could be attributed to the formation of SEI layer on the active material surface and other irreversible reaction, which is a common feature of nanostructured anodes [33, 34]. Furthermore, the charge capacity rapidly decayed to 205 mAh g^−1^ at 10th cycle and slowly decreased to 171 mAh g^−1^ at 60th (only 0.68 mAh g^−1^ capacity loss for per cycle from the 10th to 60th cycle). This result indicates that using ternary compounds with nanostructure is a potential effective alternative to improve electrochemical properties of elemental Ge for SIBs. Another important parameter of the CGO is their rate capability. As shown in Fig. 4b, the CGO nanowires manifested the reversible charge capacities of 261, 212, 164, and 130 mAh g^−1^ at current densities of 50, 100, 200, and 500 mA g^−1^, respectively. In addition, as the current density returned to 100 mA g^−1^, CGO can still deliver a high charge capacity of 175 mAh g^−1^. It is worth noting that the capacity decreases very slightly when the current densities increase from 50 to 500 mA g^−1^. This could be confirmed that ternary Ge-based compounds are a promising anode material for SIBs.

## Conclusions

In conclusion, the highly uniform CGO nanowires were prepared through a one-pot hydrothermal method, and their sodium storage electrochemical properties as anode were first explored. The as-synthesized CGO nanowires displayed an outstanding reversible capacity (306.7 mAh g^−1^ for the first cycle), a high CE (initial CE of 61.74%), a favorable cyclic performance, and a good rate capability. Ternary nanostructured compounds as anode materials not only fully utilize the intermediate products to enhance sodiation kinetics, thus providing high capacity, but also as an inert matrix to improve the cycling stability.

## Abbreviations

CE: Coulombic efficiency
CGO: CuGeO_3_
CTAB: Cetyltrimethylammonium bromide
CV: Cyclic voltammetry
EC/DMC: Ethylene carbonate/dimethyl carbonate
EVs: Electric vehicles
FEC: Fluoroethylene carbonate
Ge: Germanium
LIB: Lithium-ion battery
SEI: Solid electrolyte interphase
SEM: Scanning electron microscopy
SIBs: Sodium ion batteries
TEM: Transmission electron microscopy
XRD: X-ray diffraction
